# Syndecan-1 Regulates Vascular Smooth Muscle Cell Phenotype

**DOI:** 10.1371/journal.pone.0089824

**Published:** 2014-02-25

**Authors:** Somali Chaterji, Christoffer H. Lam, Derek S. Ho, Daniel C. Proske, Aaron B. Baker

**Affiliations:** Department of Biomedical Engineering, University of Texas at Austin, Austin, Texas, United States of America; Texas A & M, Division of Cardiology, United States of America

## Abstract

**Objective:**

We examined the role of syndecan-1 in modulating the phenotype of vascular smooth muscle cells in the context of endogenous inflammatory factors and altered microenvironments that occur in disease or injury-induced vascular remodeling.

**Methods and Results:**

Vascular smooth muscle cells (vSMCs) display a continuum of phenotypes that can be altered during vascular remodeling. While the syndecans have emerged as powerful and complex regulators of cell function, their role in controlling vSMC phenotype is unknown. Here, we isolated vSMCs from wild type (WT) and syndecan-1 knockout (S1KO) mice. Gene expression and western blotting studies indicated decreased levels of α-smooth muscle actin (α-SMA), calponin, and other vSMC-specific differentiation markers in S1KO relative to WT cells. The spread area of the S1KO cells was found to be greater than WT cells, with a corresponding increase in focal adhesion formation, Src phosphorylation, and alterations in actin cytoskeletal arrangement. In addition, S1KO led to increased S6RP phosphorylation and decreased AKT and PKC-α phosphorylation. To examine whether these changes were present *in vivo*, isolated aortae from aged WT and S1KO mice were stained for calponin. Consistent with our *in-vitro* findings, the WT mice aortae stained higher for calponin relative to S1KO. When exposed to the inflammatory cytokine TNF-α, WT vSMCs had an 80% reduction in syndecan-1 expression. Further, with TNF-α, S1KO vSMCs produced increased pro-inflammatory cytokines relative to WT. Finally, inhibition of interactions between syndecan-1 and integrins αvβ3 and αvβ5 using the inhibitory peptide synstatin appeared to have similar effects on vSMCs as knocking out syndecan-1, with decreased expression of vSMC differentiation markers and increased expression of inflammatory cytokines, receptors, and osteopontin.

**Conclusions:**

Taken together, our results support that syndecan-1 promotes vSMC differentiation and quiescence. Thus, the presence of syndecan-1 would have a protective effect against vSMC dedifferentiation and this activity is linked to interactions with integrins αvβ3 and αvβ5.

## Introduction

Vascular smooth muscle cells (vSMCs) in mature animals are highly specialized cells controlling blood vessel tone and blood pressure through their vasoregulatory activity. Mature vSMCs within adult blood vessels normally reside in a quiescent state and exhibit a low rate of proliferation, relatively low synthetic activity, and express a distinct combination of contractile proteins, ion channels, and signaling molecules. However, in cases of disease and vascular injury, vSMCs can become activated, shifting their phenotypic state through a continuum of dedifferentiated phenotypes. This can drive the vascular remodeling processes leading to restenosis and atherosclerotic plaque development.[1]

Past studies have supported that changes in the cellular microenvironment can alter the vSMC phenotype through interactions with a variety of adhesion receptors, including those of the integrin family.[2] While integrins have been extensively studied as cell adhesion receptors,[3], [4] syndecans have more recently emerged as important modulators of cellular processes.[5] Syndecans are cell surface proteoglycans consisting of an N-terminal signal peptide, an ectodomain with several consensus sequences for glycosaminoglycan attachment, a single pass transmembrane domain, and a short C-terminal cytoplasmic domain.[6] In particular, syndecans are involved in diverse cellular activities including cell adhesion,[2] growth factor signaling,[7] wound healing,[8] and skeletal muscle regeneration.[9]

Vascular smooth muscle cell growth and differentiation is mediated, in part, by the binding of various growth factors and consequent phosphorylation of receptor and non-receptor tyrosine kinases.[10] The differentiated state is characterized by a contractile state and one of the primary mechanisms of initiation of smooth muscle contraction involves the calcium/calmodulin/myosin light chain kinase-dependent phosphorylation of the myosin light chain.[11] In particular, the non-receptor tyrosine kinase Src is involved in reactive oxygen species-mediated downstream signaling via lipid rafts in vSMC membranes.[12] Src also acts as a substrate for caveolin-1[13]_ENREF_16 linking Src to calcium signaling,[14] the latter directly regulates vSMC contractility.[15] In addition, Src has been associated with modulation of the cytoskeleton.[16] Thus, Src may play a role in calcium signaling, directly regulating vSMC contractility. Direct evidence of Src's role in calcium signaling has been found in the nervous system.[17] This connection highlights the relevance of the shifted paradigm from “excitation-contraction” coupling to “excitation-transcription” coupling. In this paradigm, vSMC excitation, triggered via frequency-encoded calcium spikes, could result in CArG-mediated expression of vSMC-specific differentiation markers.[18] The expression of vSMC genes in CArG-mediated transcription occurs via the interaction of myocardin/SRF complexes and vSMC-contractile proteins containing CArG boxes. Examples of the vSMC “transcriptome” containing at least one CArG element include the majority of vSMC-specific differentiation marker genes such as smooth muscle α-actin (α-SMA), calponin, myosin heavy chain isoforms, SM22α or transgelin, telokin, and desmin.[19]

While previous studies have documented the powerful role of heparan sulfate proteoglycans in vascular biology[20], [21] our understanding of the sophistication of these regulatory processes remains limited. Specifically, the syndecan-1 gene is upregulated after vascular injury[22] and has been found to be critical for corneal and epithelial wound healing,[23] cardioprotection following myocardial infarction,[24] and in clearing triglyceride-rich lipoproteins.[25] Recent studies have shown that it has a role in regulating vascular smooth muscle cell proliferation in response to PDGF and in intimal formation following ligation injury.[26] Thus, syndecan-1 has the potential to regulate multiple aspects of vascular disease. In this study, we examined the role of the syndecan-1 in modulating the phenotype of vSMCs resulting in altered vSMC behavior. Our results support that knockout of syndecan-1 drives vSMCs toward a more dedifferentiated, activated phenotype that is resistant to differentiation by endogenous factors and demonstrates enhanced responsiveness to inflammatory stimuli.

## Materials and Methods

### Cell Maintenance and Supplements

Wild type (WT) and syndecan-1 knockout (S1KO) mice were a kind gift from Prof. Ram Sasisekharan at the Massachusetts Institute of Technology.[27] Vascular smooth muscle cells (vSMCs) were isolated from the mice using methods described earlier.[28] Isolated vSMCs were maintained in MCDB-131 media supplemented with 10% fetal bovine serum (FBS), 1% penicillin/streptomycin, and 1% l-glutamine. For optimal vSMC differentiation, media containing 1% FBS was used containing a supplement of either 30 µg/mL heparin or heparin and 5 ng/mL TGF-β1.[29] All media and supplements were obtained from Life Technologies (Grand Island, NY), unless otherwise specified. For human vSMC studies, human aortic vSMCs were obtained from Promocell (Heidelberg, Germany) and used with passage numbers ranging from 3 to 5 after receipt. For synstatin (SSTN) peptide inhibition studies, human aortic vSMCs were treated with the minimal human synstatin peptide, SSTN (93–120) (∼90% purity) with the sequence LPAGEGPKEGEAVVLPEVEPGLTAREQE (GenScript Corp., Piscataway, NJ), for 48 hours to inhibit specific syndecan-1-integrin interactions.[30], [31]

### Morphometry to Establish vSMC Phenotype

Cells were treated with control media (1% FBS), heparin, or heparin with TGF-β1 and cultured for two days. A minimum of 10 randomly selected areas (10× objective, Zeiss Axio Observer fluorescence microscope) in 4 samples of the 2 cell types (WT and S1KO) with each of the 3 treatments were imaged. These images were then analyzed by computer-assisted morphometry (Metamorph 7.0, Molecular Devices). Morphometric measurements were made of cell spread area, elliptical form factor (EFF; defined as the major axis divided by minor axis), and shape factor defined as 4π× (Area)/(Perimeter)^2^.

### In-Vitro Cell Proliferation Assay

Cell proliferation was evaluated by 5-bromo-2-deoxy-uridine (BrdU) incorporation method using a commercially available kit (Millipore, Billerica, MA). Cells were seeded at 5,000 cells/well in regular growth medium with 10% FBS in 96 well plates. BrdU was added 3 hours after cell seeding, and the assay was incubated for 24 hours after which the cells were fixed and stained with anti-BrdU antibody and read using a spectrophotometer microplate reader set at dual wavelength of 450/550 nm. In addition, an MTS assay to assess vSMC proliferation was also performed (Promega, Madison, WI). Measurements were taken in separate samples at 24 hours and 48 hours after cell seeding.

### Treatments with TNF-α and Dysfunctional Endothelial Cells to Simulate an Inflammatory Microenvironment

To mimic injury conditions such as those stemming from endothelial dysfunction following vascular interventions such as stenting, WT and S1KO vSMCs were treated with 20 ng/mL TNF-α (Peprotech, NJ). TNF-α was used as a prototype inflammatory cytokine released during vascular injury.[32], [33] Forty-eight hours after these treatments, the cells were lysed for real-time PCRs to assay the activation of the WT and S1KO vSMCs.

### Treatment of Human Aortic vSMCs with Synstatin for Mechanistic Insights into Syndecan-1's Modulation of vSMC Phenotype

To inhibit the interaction of syndecan-1 with integrins αvβ3 and αvβ5 and to determine the effect of this inhibition on vSMC phenotype, human aortic vSMCs were treated with 3 µM of the human synstatin minimal peptide in low serum medium (containing 1% FBS) and in low serum medium with heparin in order to simulate different biochemical environments. Cells were lysed after 48 hours of treatment for downstream PCRs to assay vSMC phenotype.

### Cell Lysis and Western Blotting

Cells were lysed in 1 mL of lysis buffer, which consisted of 20 mM Tris, 150 mM NaCl, 1% Triton X-100, 0.1% sodium dodecyl sulfate, 2 mM sodium orthovanadate, 2 mM phenylmethyl sulfonyl fluoride, 50 mM NaF, and a complete protease inhibitor cocktail (Roche, Nutley, NJ). After 10 minutes of incubation with the lysis buffer, the dishes were scraped with cell scrapers, and transferred into microcentrifuge tubes, sonicated, and centrifuged for 10 minutes at 14,000 g. For western blotting, the samples were run on precast NuPAGE Novex 4–12% Bis-Tris gradient gels and transferred to either poly(vinylidene difluoride) or nitrocellulose membranes. The membranes were blocked for 1 hour in StartingBlock T20 blocking buffer (Pierce, Rockford, IL) and exposed to the following primary antibodies at 4°C overnight in blocking buffer: paxillin (Abcam), syndecan-1 (Abcam), smooth muscle α-actin (Sigma), calponin (Abcam), ICAM-1 (Abcam), osteopontin (Abcam), Src (Cell Signaling), S6RP (Cell Signaling), and PKC-α (Santa Cruz Biotech), and the corresponding phospho-antibodies, p-Src, p-S6RP, and p-PKC-α (Cell Signaling). The membranes were washed with PBST and incubated at room temperature for 2 hours with horseradish peroxidase-conjugated secondaries. Detection was performed using a chemiluminescent substrate (Pierce) and imaged.

### Immunocytochemical Staining

Cells from WT and S1KO mice were seeded at densities of 3–5×10^4^ cells/cm^2^ and maintained in a proliferative medium. Cell cultures were washed with PBS, fixed with 4% paraformaldehyde for 20 minutes, followed by permeabilization with 0.2% Triton-X for ∼10 minutes at room temperature. The cultures were then blocked with 5% FBS in PBS for 1 hour at room temperature. Primary antibodies for syndecan-1 (Abcam), smooth muscle α-actin, calponin, paxillin, phospho-Src or an Alexa Fluor 594-labeled phalloidin conjugate were used for staining. Secondary antibodies conjugated to Alexa Fluor 488 or 594 (Life Technologies) at a final concentration of 5 µg/mL were used for all experiments. After extensive PBS washes, the cultures were mounted in antifade medium containing DAPI (Vector Labs). All immunostaining experiments were performed in tissue culture-treated eight well µ-slides (Ibidi, Martinsried, Germany).

### Gene Expression Analysis

Following treatments, mRNA was isolated from vSMC cultures using the RNAeasy Kit (Qiagen, Valencia, CA). The cDNA were obtained using the Taqman cDNA Reverse Transcription Kit (Life Technologies). Real time PCR was performed using the ViiA 7 Real-Time PCR System (Applied Biosystems, Foster City, CA) using a SYBR Green Master Mix (Life Technologies). All PCR results were normalized to the expression levels of GAPDH prior to further analysis. The primer pairs used for real time reverse transcription-PCR for mouse cell lysates are listed in **Table S1**.

### Immunohistochemistry

All experimental procedures and protocols used in this investigation were reviewed and approved by the Animal Care and Use Committee of the University of Texas at Austin and conformed to the Guiding Principles in the Care and Use of Animals of the American Physiological Society and the NIH Guide for the Care and Use of Laboratory Animals. Age-normalized (24 months of age) WT and S1KO mice were euthanized with CO_2_ (n = 5). Aortae were removed, frozen in liquid nitrogen cooled isopentane and stored at −80°C. The descending thoracic aorta was used for mounting and sectioned in 8 µm sections using a cryotome (Leica). The sections were fixed in acetone at −20°C and stored at −80°C until staining. Immunostaining was done for calponin with a rabbit primary (Abcam) and Alexa Fluor 594 (Life Technologies) as secondary. Sections were counterstained with DAPI-containing mounting medium and imaged using a Zeiss fluorescent microscope.

### Statistical Analysis

All results are shown as mean ± standard error of the mean (SEM). A two-tailed Student's t-test was used to make comparisons in data with only two groups with p<0.05 counted as statistically significant. ANOVA was used for multiple comparisons and Tukey's post-hoc testing was used to assess differences between groups. *Indicates a statistically significant difference between WT and S1KO groups (p<0.05). For all other comparisons, the signage indicates statistically significant difference for the specified treatment groups.

## Results

### Loss of Syndecan-1 Altered vSMC Phenotype with Enhanced Proliferation and Distinct Morphological Characteristics

**Figure 1:**
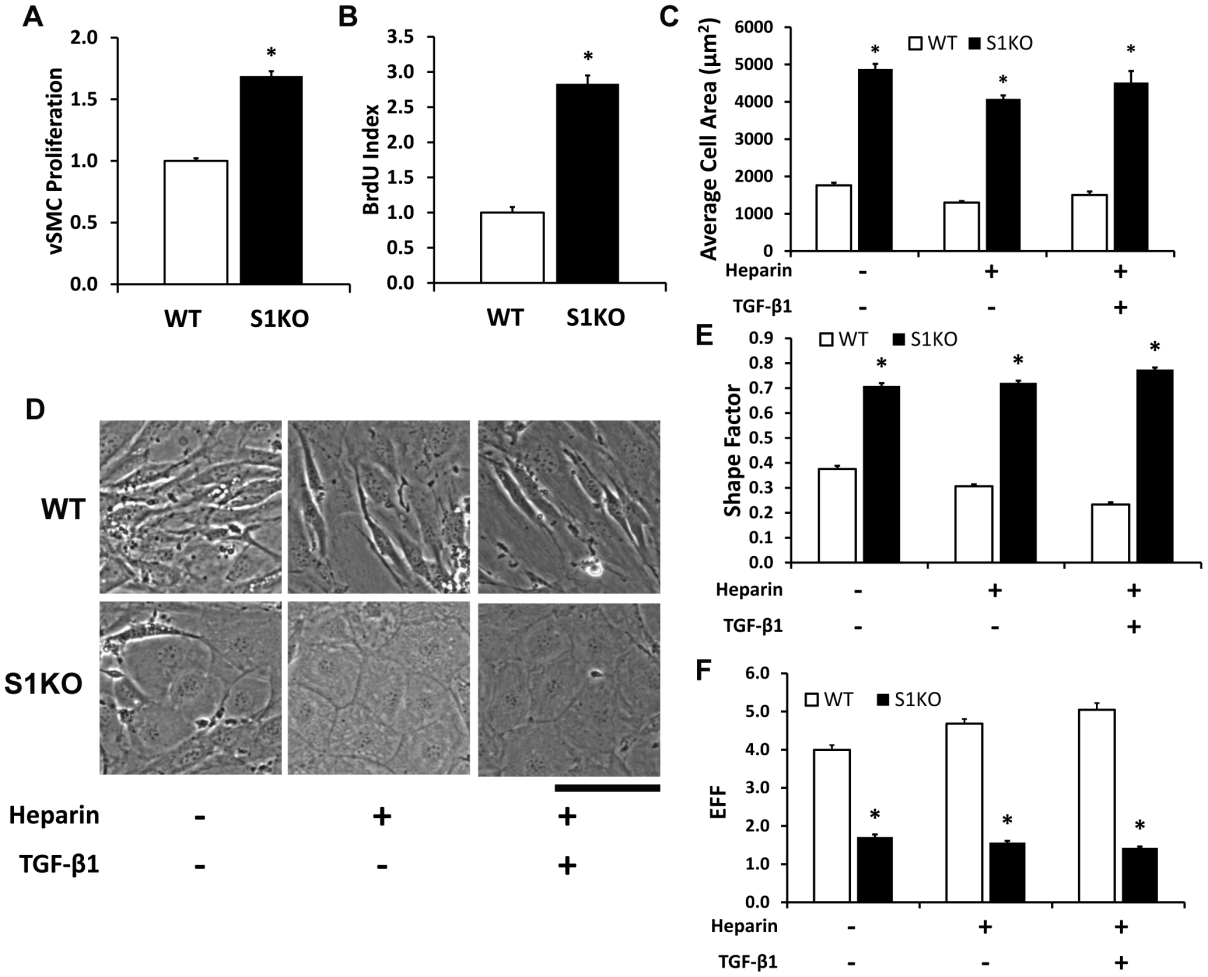
Syndecan-1 knockout increases vSMC proliferation and induces a more spread adherent cell morphology. (A) Cell proliferation measured using an MTS assay demonstrated faster growth in syndecan-1 knockout (S1KO) vSMCs versus wild type (WT) vSMCs. (B) DNA synthesis in S1KO and WT cell lines, as indicated by the BrdU index. (C) Altered morphology of S1KO vSMCs when subjected to changes in the biochemical environment, specifically control (1% FBS), medium containing 30 µg/mL heparin, and medium containing 30 µg/mL heparin and 5 ng/mL TGF-β1. (D) Cell area after spreading was smaller for WT vSMCs in comparison to S1KO vSMCs. (E) Shape factor determinations indicated that S1KO vSMCs were more circular than WT vSMCs. (F) Elliptical form factor (EFF) determinations indicated that S1KO vSMCs were shorter and wider than their WT counterparts. Scale bar is 100 µm. *Statistically significant difference with WT group under similar culture conditions (p<0.05).

We isolated vSMC from WT and S1KO mice and knockout of syndecan-1 was confirmed using immunostaining and western blotting (**Figure S1**). Under high serum conditions (10% FBS), the S1KO vSMCs proliferated more rapidly than WT vSMCs, as measured using MTS assays (**Figure 1A**) and through BrdU incorporation assays (∼2.8 fold increase, **Figure 1B**). To stimulate vSMC differentiation, the cells were treated with heparin, or, with heparin and 5 ng/mL TGF-β1. Both heparin and TGF-β1 are known to drive vSMCs to a differentiated state in human aortic and coronary vSMCs.[29] Syndecan-1 knockout vSMCs had a markedly altered morphology in comparison to WT vSMCs (**Figure 1C**), with a greater adhered area (**Figure 1D**), higher circularity, and lower elliptical form factor under the various treatment conditions (**Figure 1E**). Histograms for cell area indicated that total spread cell area had increased in S1KO cells for all treatments (**Figure S2**). To assess potential compensatory mechanisms in the cells due to syndecan-1 knockout, PCRs were performed for other the syndecans. The results indicated increased mRNA levels of syndecans-2 and -4 in S1KO vSMCs in comparison to WT vSMCs under most of the treatment conditions (**Figure S3**).

### Syndecan-1 Knockout Decreased Gene Expression of vSMC Differentiation Markers and Increased Tissue Factor Expression

**Figure 2:**
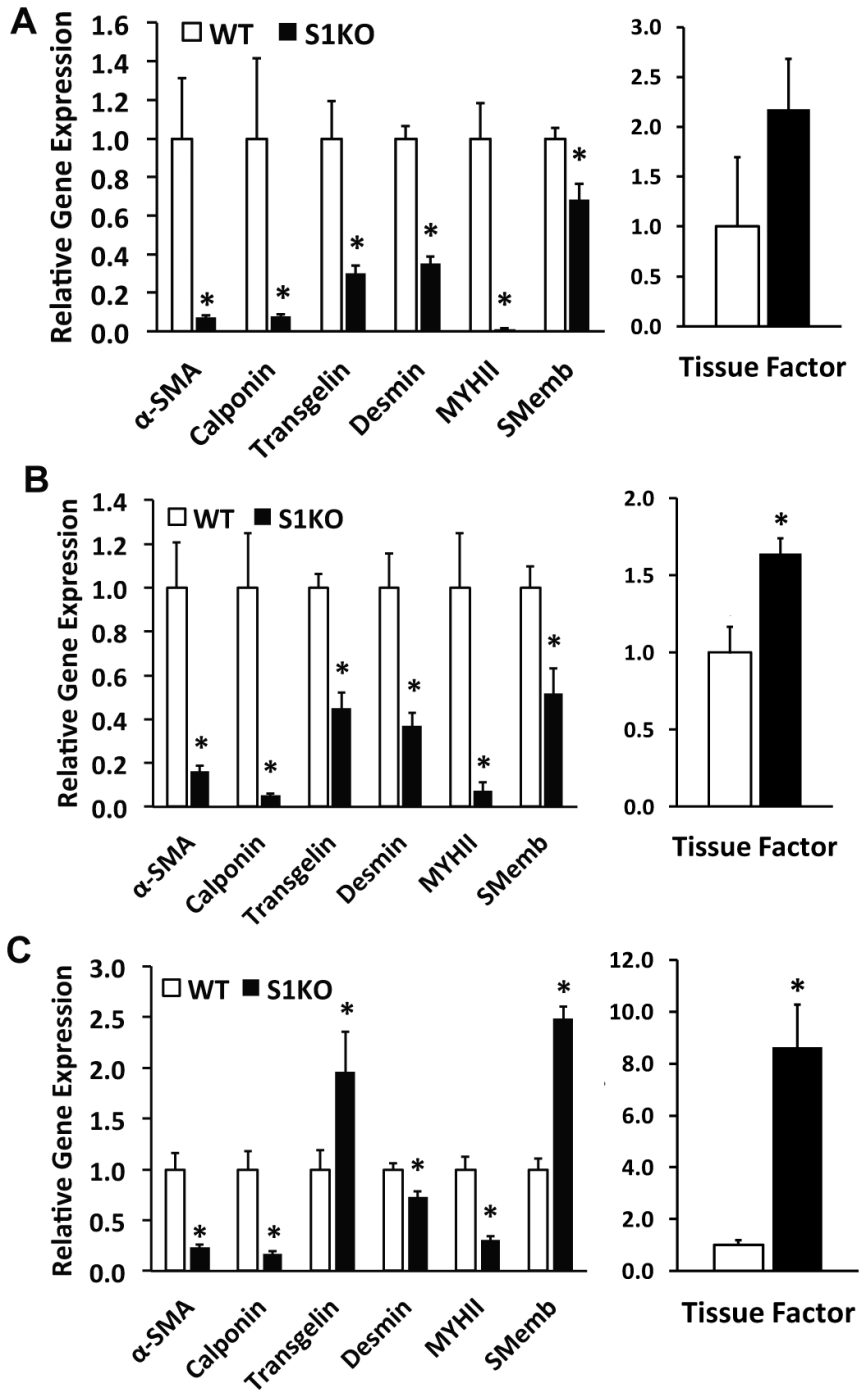
Syndecan-1 knockout leads to loss of gene expression of mature vascular smooth muscle cell (vSMC) markers. Real time PCR analyses of vSMC differentiation-related markers and tissue factor in vSMCs under the following treatments: (A) control (1% FBS), (B) medium containing 30 µg/mL heparin, and (C) medium containing 30 µg/mL heparin and 5 ng/mL TGF-β1. These analyses indicated that vSMCs were more differentiated and expressed higher levels of vSMC-specific differentiation markers in WT vSMCs relative to S1KO vSMCs. In addition, SMemb, the embryonic form of myosin heavy chain, was higher in S1KO vSMCs after treatment with heparin and TGF-β1. *Statistically significant difference with WT group under similar culture conditions (p<0.05).

The cells were treated with control media (1% FBS), heparin, or, heparin with TGF-β1, for 48 hours, and then examined for the gene expression of vSMC-specific markers using real time PCR. Under control and heparin-treatment conditions, the gene expression for differentiation markers for mature vSMCs was dramatically lowered in S1KO vSMC, including α-SMA, calponin, desmin, and myosin heavy chain 11 (MYH11; **Figures 2A and 2B**). Interestingly, the levels of non-muscle myosin (SMemb) and transgelin were also lowered under these treatments. After treatment with heparin and TGF-β1, the differentiation markers were lowered in S1KO cells and both SMemb and transgelin were markedly increased (**Figure 2C**). Tissue factor gene expression was enhanced in S1KO cells under all the conditions tested (**Figure 2A–C**).

### Syndecan-1 Knockout Leads to a Reduction in α-SMA and Calponin Protein Expression cultured vSMCs

**Figure 3:**
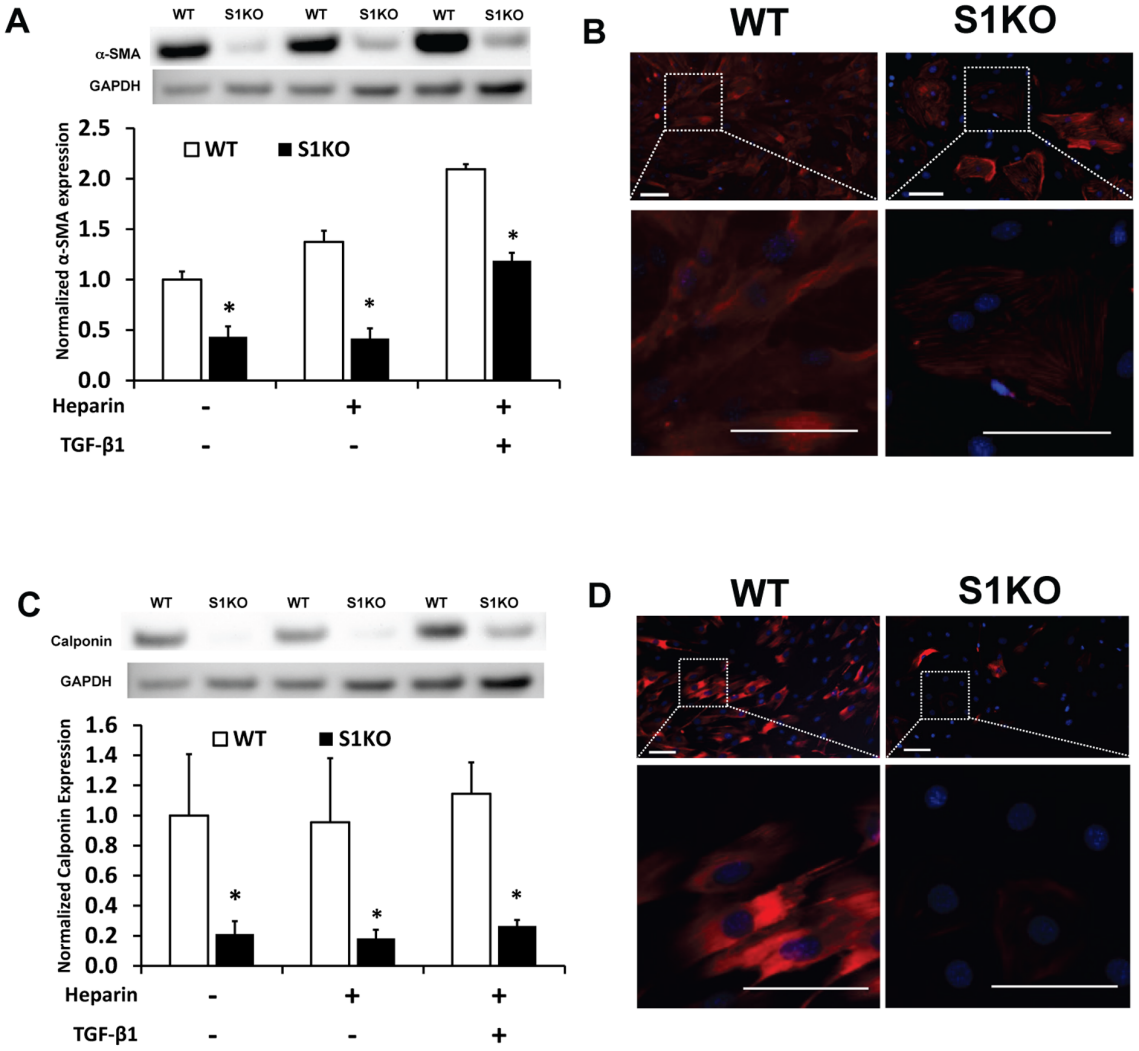
Measurement of protein levels of α-SMA and calponin in cultured vSMCs confirmed the upregulation of α-SMA and calponin in WT vSMCs. (A, C) Western blotting for α-SMA and calponin in WT and S1KO cells after 48 hours of the shown treatments. (B, D) Immunostaining for α-SMA and calponin demonstrated higher expression in WT vSMCs versus S1KO vSMCs. Scale bars are 100 microns in length. *Statistically significant difference with WT group under similar culture conditions (p<0.05).

To confirm the potent effects of syndecan-1 knockout at the protein level we examined α-SMA and calponin expression and localization in the cells. Western blotting to control, heparin, or heparin and TGF-β1 treated cells demonstrated lower protein expression levels of these differentiation markers in S1KO vSMCs (**Figures 3A and 3C**). While α-SMA expression increased progressively with the differentiation treatments for WT vSMCs, levels increased for calponin only with combination treatment with heparin and TGF-β1. Immunocytochemical analyses for α-SMA and calponin demonstrated qualitatively higher staining in WT vSMCs, in addition to varied morphology of these contractile vSMC-specific elements (**Figures 3B and 3D**).

### Loss of Syndecan-1 Altered Actin Cytoskeletal Arrangement and Focal Adhesion Complex Organization

**Figure 4:**
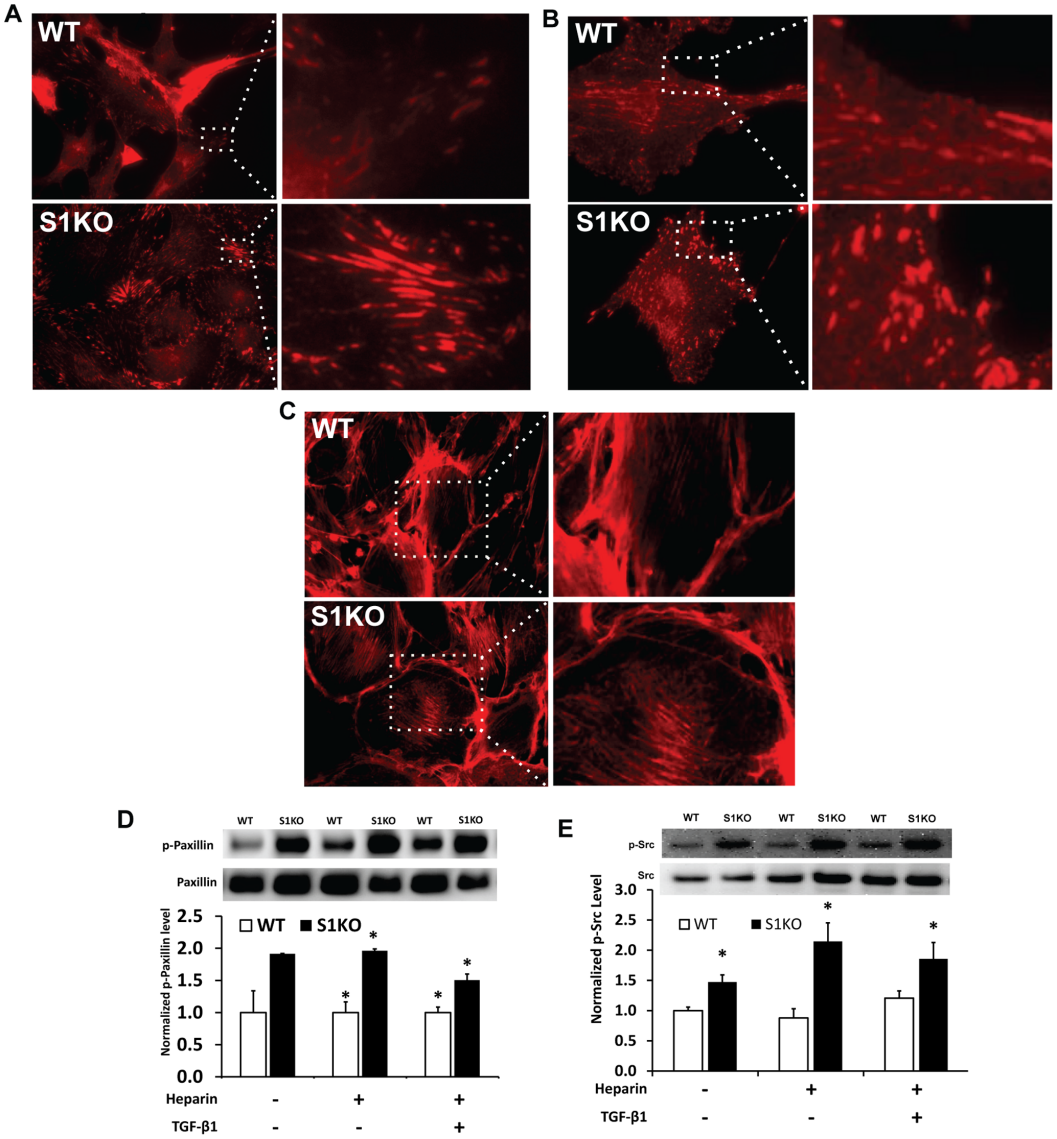
Immunocytochemical analyses of the actin cytoskeleton and focal adhesions in WT and S1KO vSMCs. (A) Paxillin in focal adhesions was more abundant and localized in large plaques near the periphery in S1KO vSMCs relative to WT. (B) Increased p-Src levels in WT relative to S1KO were observed in all media with representative images of p-Src expressed by WT and S1KO vSMCs. (C) Actin stress fibers were found to be centralized toward the nucleus in S1KO vSMCs relative to WT. (D) Increased p-paxillin levels in S1KO vSMCs was indicated by Western blotting. For all western blots, cell lysates were obtained from confluent vSMCs that were treated with control medium or with heparin or with heparin and TGF-β1 for 48 hours prior to lysis. In all Western blots, the expression levels of the target protein for S1KO vSMCs were normalized to those for WT vSMCs treated with control medium. *Statistically significant difference with WT group under similar culture conditions (p<0.05).

Based on the changes in cell morphology, the cytoskeletal and focal adhesion organization of WT and S1KO vSMCs was examined. In S1KO vSMCs, paxillin in focal adhesions appeared to be localized in larger plaques at the periphery of the cell while being found distributed more diffusely in WT cells (**Figure 4A**). This finding corresponded to a similar distribution of phospho-Src (**Figure 4B**). There was also a marked increase in the formation of actin stress fibers in the S1KO cells with a particular enhancement in perinuclear actin stress fibers (**Figure 4C**). For all differentiation treatments, there was a significant increase in paxillin phosphorylation in S1KO relative to WT vSMCs, as evidenced by western blotting (**Figure 4D**). Further, the phosphorylation of Src was higher for S1KO than for WT vSMCs under all of the culture conditions (**Figure 4E**).

### Syndecan-1 Knockout Altered Cell Survival and Proliferation-Related Signaling Pathways

**Figure 5:**
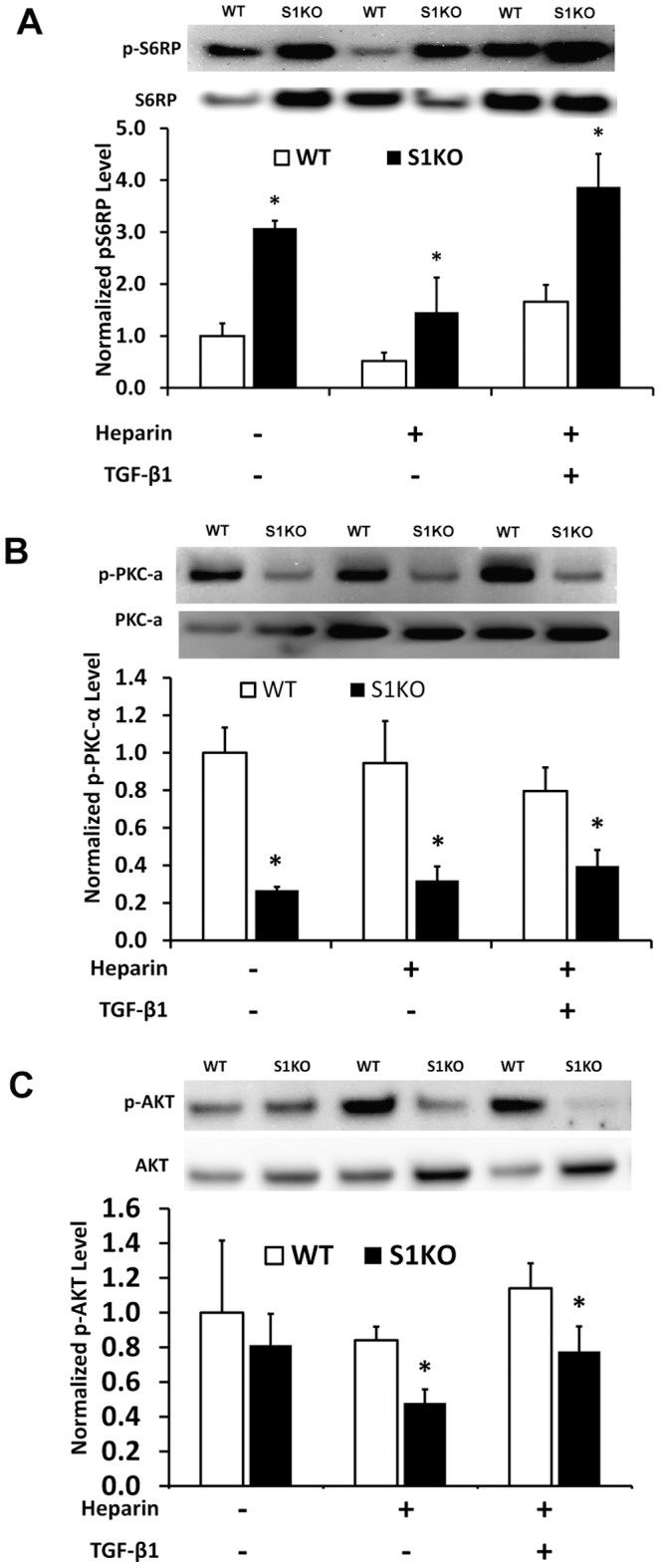
Western blotting analysis of intracellular signaling pathways in WT and S1KO vascular smooth muscle cells (vSMCs). For all western blots, cell lysates were obtained from vSMCs that were treated with 1% FBS, heparin or with heparin and TGF-β1 for 48 hours prior to cell lysis. (A) Western blotting for phospho-S6RP and total S6RP. (B) Immunoblotting analyses for phosphorylated PKC-α and total PKC-α. (C) Western blotting indicated reduced phospho-AKT in S1KO vSMCs versus WT vSMCs in heparin and heparin/TGF-β1 treated cells. In all quantification analyses, expression levels of the target protein for S1KO vSMCs were normalized to those for WT vSMCs treated with control culture medium. *Statistically significant difference with WT group under similar culture conditions (p<0.05).

The phosphorylation level of S6 ribosomal protein (S6RP) is a downstream marker of the PI3K/AKT/mTOR pathway, which a key pathway for vSMC survival and apoptosis resistance in vascular disease. The phosphorylation of S6RP was assayed after 48 hours of treatment with heparin or heparin and TGF-β1. Phosphorylation of S6RP was increased by more than two-fold in S1KO vSMCs under all conditions (**Figure 5A**). There was also a decrease in AKT phosphorylation (p-Ser^473^) in S1KO vSMCs (**Figure 5B**). In addition, decreased PKC-α (p-Ser^657^) phosphorylation in S1KO vSMCs was observed in comparison to WT (**Figure 5C**).

### Measurement of vSMC Differentiation Markers in the Aorta of WT and Syndecan-1 KO Mice

**Figure 6:**
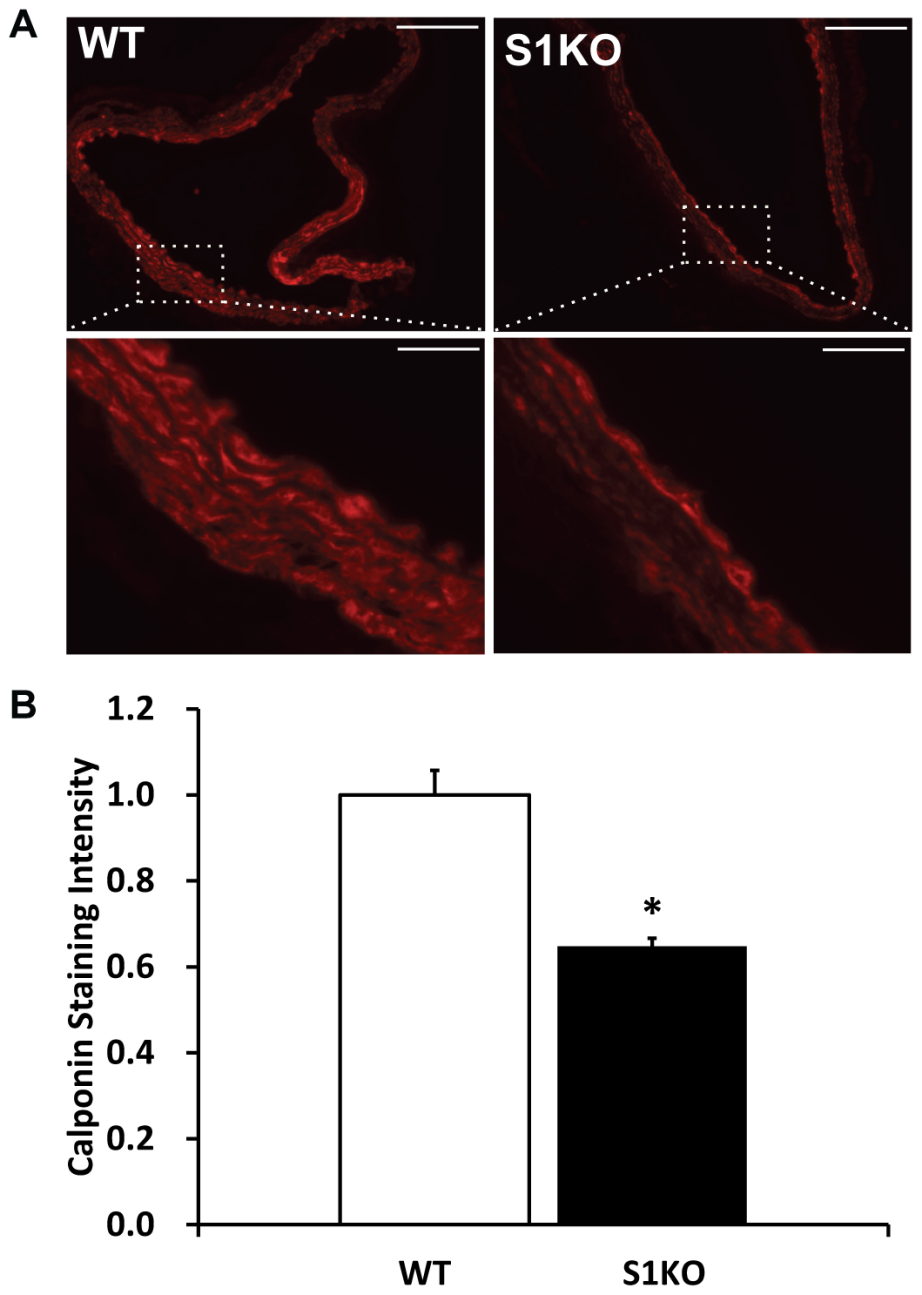
Reduced expression of calponin by aged syndecan-1 knockout (S1KO) mouse aortae relative to old wild type (WT) mice aorta. (A) Immunohistochemical staining for calponin in the descending aorta harvested from the mice. (B) Morphometric quantification of calponin staining in the arteries (n = 5). All images were taken at the same length of exposure and calponin staining relative intensities were quantified. The intensities for S1KO mouse descending aorta were normalized relative to WT. Scale bars are 100 microns in length. *Statistically significant difference with WT (p<0.05).

Descending aortae from aged WT and S1KO mice were harvested and stained them for α-SMA and calponin. In our studies, there were variations in α-SMA expression by WT and S1KO aortae, with a trend toward a decrease in α-SMA in the S1KO vSMCs but these were not statistically significant (data not shown). However, the S1KO vSMCs had a lower expression of calponin in samples from descending aorta of S1KO mice (**Figure 6**). α-SMA appears early during vSMC differentiation[34] and the alteration of its expression with vSMC phenotypic modulation is gradual while calponin appears later in development and its expression is dramatically reduced upon phenotypic changes.[35]

### Inflammatory Responsiveness to TNF-α is increased in S1KO vSMCs

**Figure 7:**
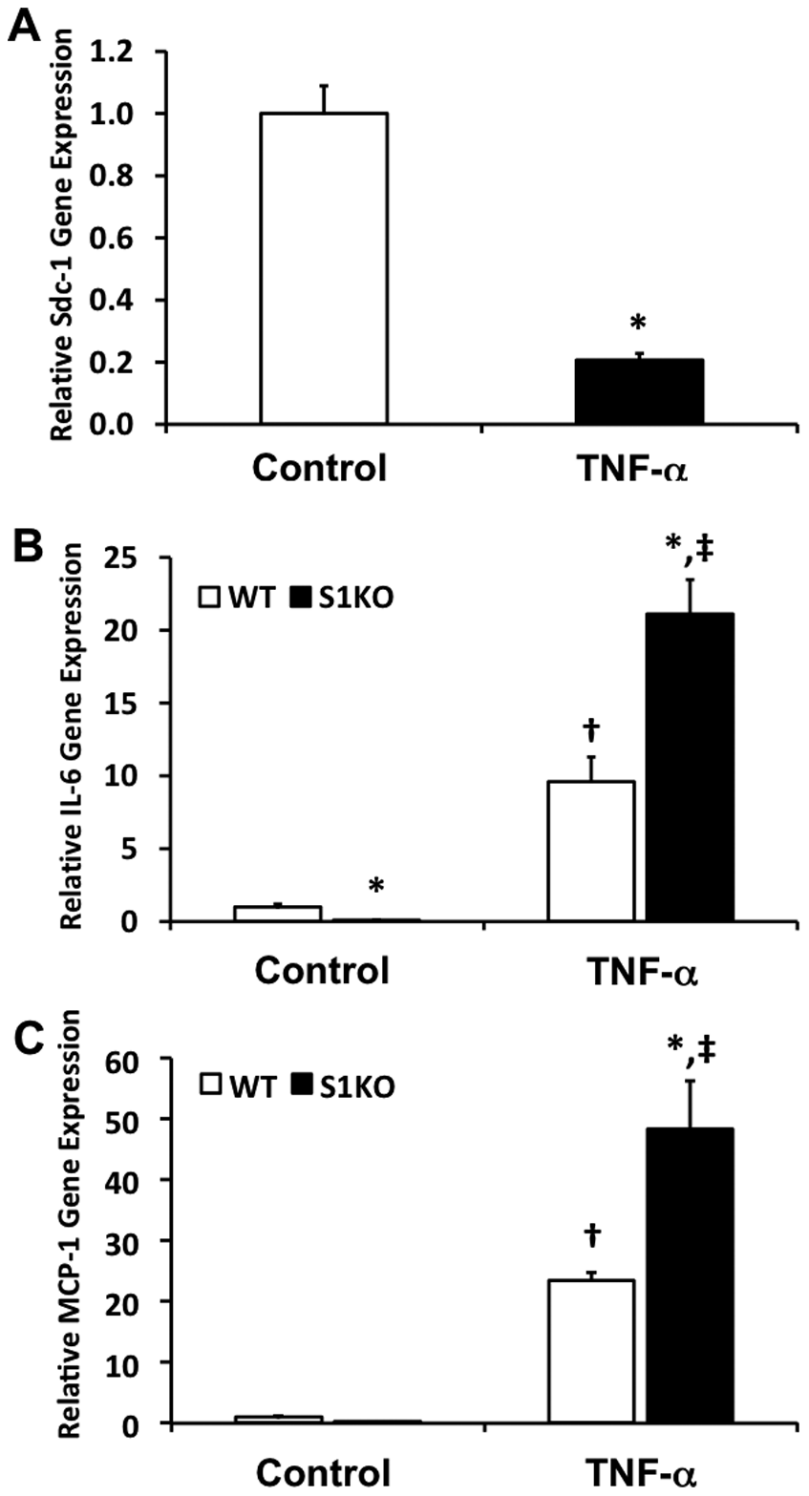
Treatment of vascular smooth muscle cells (vSMCs) with TNF-α alters expression of syndecan-1 (sdc-1) and the absence of syndecan-1 in syndecan-1 knockout (S1KO) increases the expression of inflammatory cytokines by vSMCs. The cells were treated with 20/mL TNF-α for 48 hours. (A) Treatment of WT mouse cells with TNF-α reduces gene expression of sdc-1. (B) Baseline expression of IL-6 was lower in S1KO vSMCs but higher after stimulation with TNF-α. (C) Higher MCP-1 gene expression in S1KO vSMCs relative to WT vSMCs after stimulation with TNF-α. *Statistically significant difference with WT cell group under similar culture conditions. †Statistically significant difference with non-TNF-α treated WT cell group. ‡Statistically significant difference with non-TNF-α treated S1KO cell group.

Vascular injury or atherosclerotic disease processes can drive inflammation with the artery. We stimulated vSMCs with TNF-α and examined the ensuing expression of inflammatory cytokines. Interestingly, treatment with TNF-α decreased syndecan-1 expression in WT vSMCs by over 80% (**Figure 7A**). Further, S1KO vSMCs had a two-fold increase in expression of IL-6 and MCP-1 following TNF-α treatment (**Figure 7B and 7C**). Together these results support that S1KO vSMCs are more responsive to inflammatory stimulation and that inflammatory stimuli can reduce sdc-1 expression.

### Mechanistic Effect of Blocking the Interaction of Syndecan-1 with Integrins αvβ3 and αvβ5

**Figure 8:**
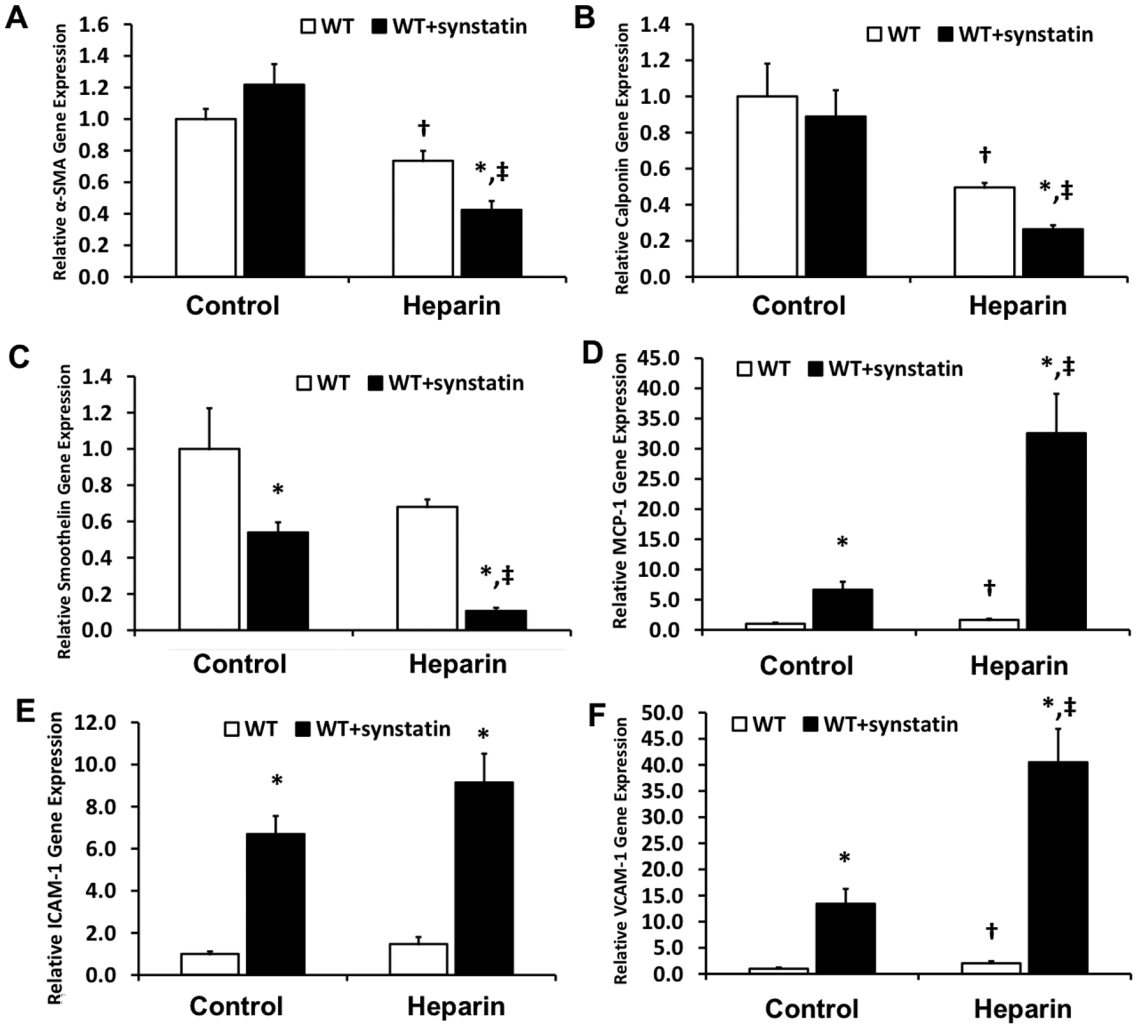
Inhibition of the interactions of syndecan-1 with integrins αvβ3 and αvβ5 was inhibited with the synstatin and gene or protein expression was measured after 48 hours. (A–C) Decreased expression of vSMC-specific differentiation markers; (D–F) Increased expression of inflammatory cytokine, MCP-1, and, adhesion receptors, ICAM-1 and VCAM-1.

**Figure 9:**
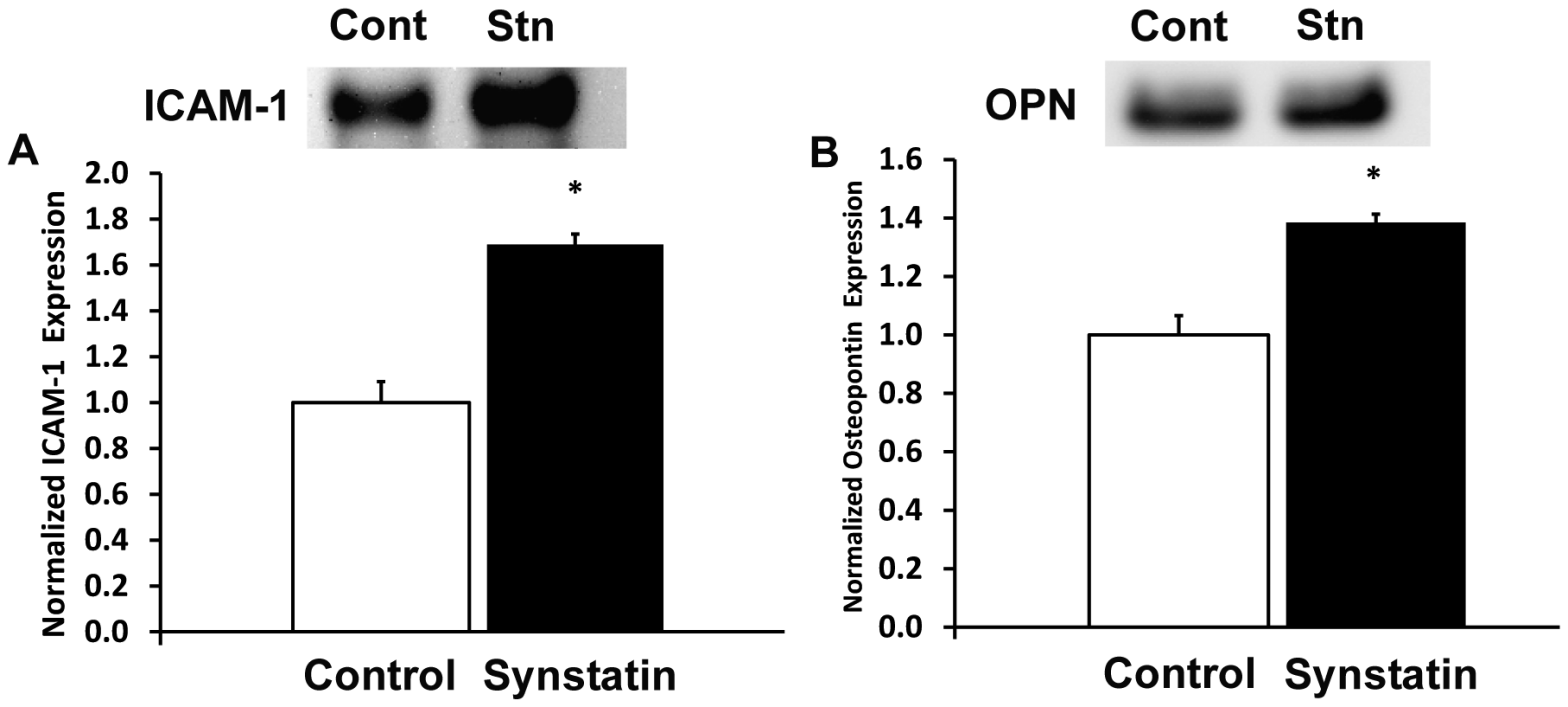
Western blotting for increased expression of ICAM-1 and osteopontin. *Statistically significant difference with WT group under similar culture conditions. †Statistically significant difference with non-heparin treated WT group. ‡Statistically significant differences with non-heparin treated WT/synstatin group.

We next inhibited the interactions of sdc-1 and integrins αvβ3 and αvβ5 using the synstatin inhibitory peptide.[36] Treatment with the minimal synstatin peptide inhibited vSMC differentiation leading to decreased expression of α-SMA, calponin, and smoothelin (**Figure 8A–C**). In addition, synstatin treatment increased the expression for inflammatory cytokines and adhesion receptors such as MCP-1, ICAM-1 and VCAM-1, and osteopontin (**Figure 8D–F**). Increased expression of ICAM-1 and osteopontin was also verified using western blotting, confirming the increased expression of these inflammatory proteins (**Figure 9**). Synstatin addition to the 1% FBS (control) vSMC medium enhanced the expression of inflammatory cell markers. However, in the presence of heparin, the vSMC activation stemming from synstatin addition was more dramatic, with an approximately six-fold increase in MCP-1 and a three-fold increase in VCAM-1 expression. Thus, this indicates that heparin may be facilitating synstatin-mediated inhibition, adversely affecting vSMC phenotype.

## Discussion

In this study, we demonstrate that syndecan-1 is a regulator of vSMC phenotype and contractile state. Taken together, our findings support the concept that syndecan-1 maintains the differentiated state of vSMCs and consequently its expression may have a vasculoprotective effect in the context of vascular disease and injury. Alterations in vSMC phenotype play a key role in vascular remodeling in several prominent vascular disease states, including atherosclerosis, hypertension, and restenosis.[37] In many of these disease states, vSMCs transition from a contractile, differentiated phenotype toward a dedifferentiated state with enhanced proliferation, expression of extracellular matrix, and a concomitant downregulation of vSMC-specific differentiation markers.[1] Previous studies have shown that vSMC phenotype can be modulated by an assortment of pathophysiologic conditions, recapitulated *in vitro*, such as altering the scaffold topography or stiffness,[38] mechanical stimulation,[39]–[41] biochemical stimulation,[29] or tissue geometry.[42] Syndecan-1 is involved in both the response to soluble biochemical factors and in cell interactions with extracellular matrix.[43] A prior study by Fukai *et al*. has shown that Sdc-1 knockout (S1KO) vSMCs proliferate faster and that S1KO mice have increased intimal formation in response to ligation injury.[44] While dedifferentiation of vSMCs has been linked to increased proliferation, these two processes are regulated in a complex manner.[45], [46] For example, several studies have shown that FGF-2 can modulate vSMC differentiation independent of proliferation.[47]–[49] Our findings extend previous work by identifying that syndecan-1 expression maintains the mature phenotype in vSMCs and influences multiple downstream pathways relating to cell survival, calcification, and thrombosis.

A major finding of our study was that the loss of syndecan-1 leads to a reduction in the expression of differentiation markers in different biochemical environments with an accompanying increase in tissue factor gene expression. Phenotypic modulation of vSMCs is characterized by the downregulation of differentiation marker genes and upregulation of genes relating to inflammation, proliferation, and the extracellular matrix.[50] Many vSMC-specific differentiation factors are downregulated when the vSMC phenotype is altered in the context of disease or after vascular injury.[1] A prime functional role of vSMCs in the artery is the control of vasomotor tone and, consequently, many of the markers of the differentiated state are key components of the contractile apparatus, including calponin and α-SMA. In the differentiation continuum, α-SMA is expressed in early stages, with calponin being expressed later on, and particular myosin heavy chain isoforms, including MYH11, expressed in mature vSMCs.[1] In contrast, SMemb is a marker of embryonic vSMCs and is increased in vSMCs in the neoinitima.[51] Our study examined the expression of these markers in vSMCs under low serum and after stimulating the cells with heparin or heparin/TGF-β. Both of these treatments are known to drive vSMCs to a more mature phenotype.[52], [53] In our study, the markers for mature vSMCs were reduced in syndecan-1 knockout cells. However, only under treatment with heparin/TGF-β was SMemb increased over the level of gene expression in WT cells.

In our study, the loss of syndecan-1 led to a marked dysregulation of contractile and proteins involved in cell adhesion. Additionally, syndecan-1 knockout led to decreased protein levels of both calponin and α-SMA. Calponin is a major component of the smooth muscle thin filament and localizes in the vSMC contractile apparatus where it binds to actin with high affinity, modulating actin-myosin interactions that are responsible for contraction.[54] It is also known to bind to calmodulin, myosin, and desmin in the contractile apparatus.[55] Calponin stabilizes actin stress fibers and its downregulation destabilizes the cytoskeleton *in vivo* and *in vitro*.[56], [57] As the contractile state is often linked to the mature vSMC phenotype, this finding is consistent with our studies on gene expression in the syndecan-1 knockout cells. In contrast, syndecan-1 knockout increased focal adhesion formation with enhanced presence of phosphorylated paxillin and Src. Src is a regulator of focal adhesion formation[58] and is stimulated in vSMCs in response to oxidative stress and other inflammatory stimuli.[59], [60] Src kinases are known to interact with the cytoplasmic domains of syndecans via their SH2 domains and alter the cellular cytoskeleton and cell motility via regulation of lamellipodial dynamics.[61], [62] Thus, enhanced Src phosphorylation in syndecan-1 knockout vSMCs may support the increased proliferation, observed by others and confirmed in our study, as well as contributing to the more dedifferentated state observed in these cells.

Vascular injury is known to lead to endothelial denudation and direct exposure of vSMCs to blood flow. Our study identified an eight-fold increase in tissue factor gene expression in syndecan-1 knockout vSMCs after treatment with heparin and TGF-β1. Tissue factor is an initiator of the extrinsic coagulation pathway.[63] Tissue factor binds factor VIIa (FVIIa) to form a complex that leads to activation of factors IX and X, resulting in thrombin generation and fibrin crosslinking.[64] Tissue factor is expressed by medial vSMCs and adventitial fibroblasts and exposed to blood during vascular injury.[65] Increased expression of TF is also present in atherosclerotic plaques.[66] In addition to its role in coagulation, the TF:FVIIa complex activates the protease-activated receptor 2 (PAR-2) leading to induction of pro-inflammatory signals that have been associated with increased proliferation and migration in vSMCs. These signals enhance the production of pro-inflammatory cytokines resulting in immune cell recruitment within atherosclerotic plaques.[67] Syndecan-1 and heparanase have been linked to synergistic activities in cancer. Heparanase acts to cleave the heparan sulfate chains on syndecan-1 leading to enhanced shedding.[68] Recent studies have also shown that heparanase overexpression leads to enhanced TF expression[69] and thrombosis in response to vascular injury.[21] Thus, our findings would suggest that loss of syndecan-1, in the context of TGF-β1 stimulation, leads to an increase in TF expression and thus may act to enhance the risk of arteriothrombosis.

In vSMCs lacking syndecan-1, we found a marked decrease in PKC-α and AKT phosphorylation. Conversely, we also found an increase in S6RP phosphorylation that increased further in response to TGF-β1 treatment. Opposing regulation of AKT and S6RP was also found for syndecan-4 null cells.[70], [71] In addition, syndecan-4 has been found to regulate mTORC2-dependent AKT activation.[70]–[72] Activation of AKT has been associated with the differentiated phenotype of vSMCs[73], [74] and sustained AKT activation is required for vSMC differentiated marker expression.[75] Thus, the downregulation of AKT in syndecan-1 vSMCs is consistent with the dedifferentiated phenotypic state of these cells. While AKT is commonly associated with the increased phosphorylation of S6RP,[76] our results suggest that syndecan-1 may regulate AKT through mTORC2, similar to syndecan-4.[77] Thus, it is possible that syndecan-1 limits the mTORC1 activity with subsequent downregulation of S6RP phosphorylation, but does not inhibit mTORC2 activity, leading to increased AKT and PKC-α phosphorylation.

The formation of atherosclerotic plaques and neointimal hyperplasia following percutaneous interventions is associated with increased levels of TNF-α.[78], [79] In *in-vitro* studies, TNF-α can stimulate migration in vSMCs[80], [81] and also induces the production and release of numerous inflammatory factors, including various members of the interleukin family and matrix metalloproteases.[81]–[83] Our study found that TNF-α decreased syndecan-1 expression in WT vSMCs and that knockout of syndecan-1 induced increased levels of IL-6 and MCP-1 on treatment with heparin. In addition, inhibition of syndecan-1 interactions with integrins αvβ3/αvβ5 using the synstatin peptide led to increased expression of inflammatory adhesion molecules, cytokines, and osteopontin in vSMCs. Syndecan-1 interacts with αvβ3 and αvβ5 through a region on the extracellular domain.[20], [21] Previous studies have shown that integrins αvβ3 and αvβ5 are upregulated in vascular injury and may be responsible for enhanced vSMC migration.[22] The synergistic effects of integrins and syndecans have been reported in literature in different cell types, including vascular cells.[23], [24] Our study suggests that the interaction of syndecan-1 and these integrins is necessary for the anti-inflammatory and atheroprotective effects of syndecan-1 expression in vSMCs. This is supported by the increased expression of inflammatory markers and osteopontin when these specific interactions are blocked by synstatin. In addition, osteopontin is associated with the migration of vSMCs[84] and is considered an osteogenic molecule that would support vascular calcification in atherosclerosis.[85] Thus, the interactions of syndecan-1 with integrins αvβ3 and αvβ5 appear to be critical to its activity in suppressing the inflammatory and osteogenic state in vSMCs.

In conclusion, our results indicate that syndecan-1 is a potent modulator of vSMC phenotype that can potentially regulate multiple aspects of vSMC biology, including contraction, inflammation, thrombosis, and calcification. We demonstrated that syndecan-1 regulates vSMC expression of differentiation markers, cytoskeletal arrangement, and intracellular signaling through the AKT, PKC-α, and S6RP pathways. Our findings provide insights into a novel class of cell-surface receptors responsible for vSMC differentiation and suggest that the preservation or enhancement of syndecan-1 expression may be a potential therapeutic target capable of altering the course of in-stent restenosis and atherosclerosis. A potential strategy for increasing cell surface levels of syndecan-1 is the inhibition of matrix metalloprotease or heparanase mediated-shedding of syndecans through specific inhibitors to these enzymes. In addition, we have recently explored using syndecans delivered in liposomal carriers as a means for enhancing revascularization in ischemic disease and a similar strategy may provide a potential means to enhance the presence of syndecan-1 on the cell surface.[86], [87]

## Supporting Information

Figure S1 (A) Western blotting for syndecan-1 confirmed the loss of syndecan-1 in syndecan-1 knockout (S1KO) vSMC; syndecan-1 was detected at a molecular weight of approximately 100 kDa. (B) Immunohistochemical staining for syndecan-1 in wild type (WT) and S1KO vSMCs. Scale bar is 100 microns in length.
(TIF)

Figure S2 Histograms of cell areas for (A) 1F-treated vSMCs (control cells), (B) heparin-treated vSMCs, and (C) heparin and TGF-β1-treated vSMCs. Binning in the x-axis is area in µm^2^.
(TIF)

Figure S3 Gene expression for syndecan-2 (sdc-2) and syndecan-4 (sdc-4) in vascular smooth muscle cells (vSMCs) isolated from wild type (WT) and syndecan-1 knockout (S1KO) mice. Cells were treated with (A) control (1% FBS), (B) medium containing 30 µg/mL heparin, and (C) medium containing 30 µg/mL heparin and 5 ng/mL TGF-β1. *Statistically significant difference with WT group under similar culture conditions (p<0.05).
(TIF)

Table S1 (DOCX)
