# Supplementary figures and images for: Syndecan-1 Regulates Vascular Smooth Muscle Cell Phenotype
Source: PLoS One. 2014 Feb 25;9(2):e89824. doi: 10.1371/journal.pone.0089824 (PMC3934950; doi:10.1371/journal.pone.0089824)

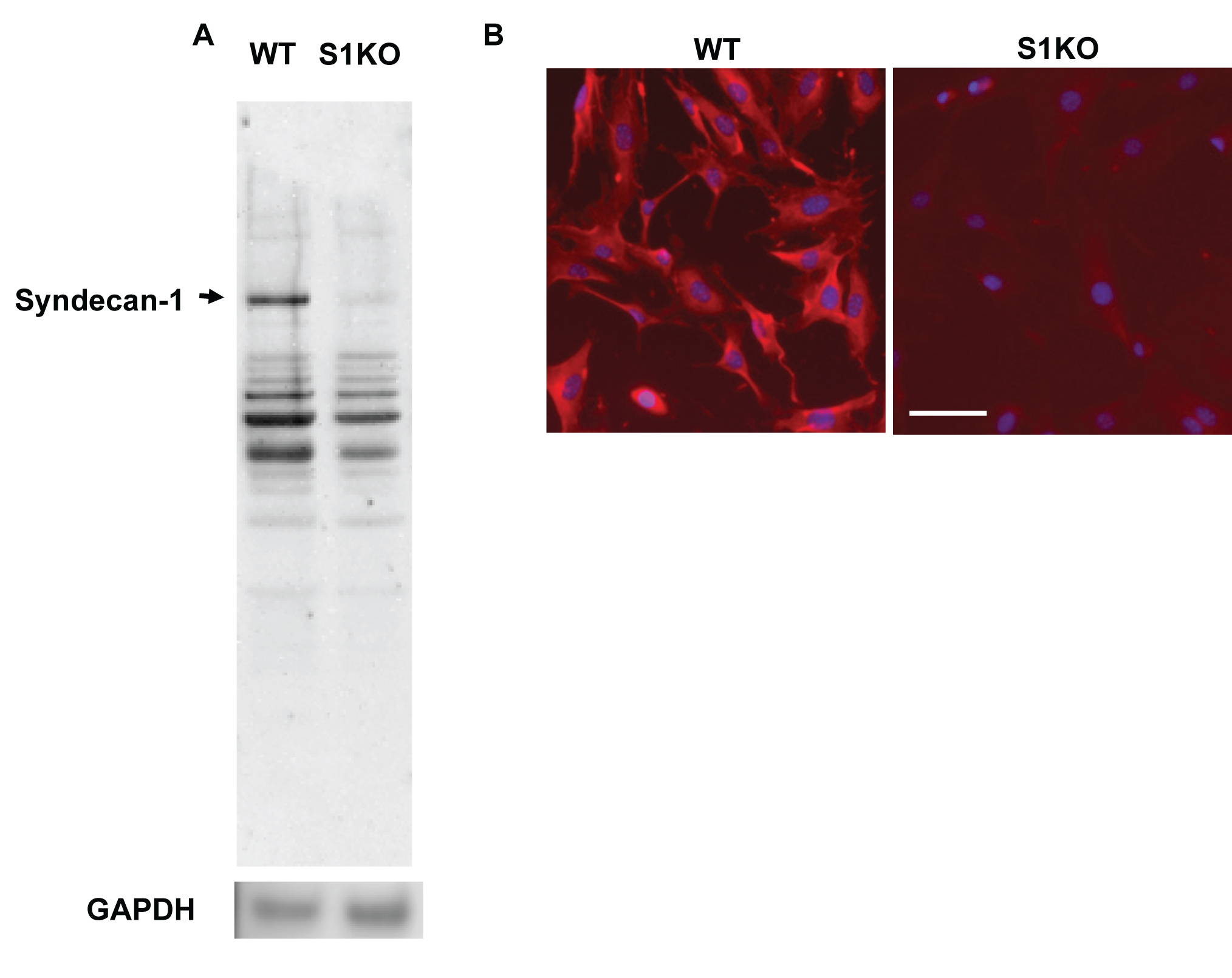

Supplement: Figure S1 — (A) Western blotting for syndecan-1 confirmed the loss of syndecan-1 in syndecan-1 knockout (S1KO) vSMC; syndecan-1 was detected at a molecular weight of approximately 100 kDa. (B) Immunohistochemical staining for syndecan-1 in wild type (WT) and S1KO vSMCs. Scale bar is 100 microns in length. (TIF) [file pone.0089824.s001.tif]

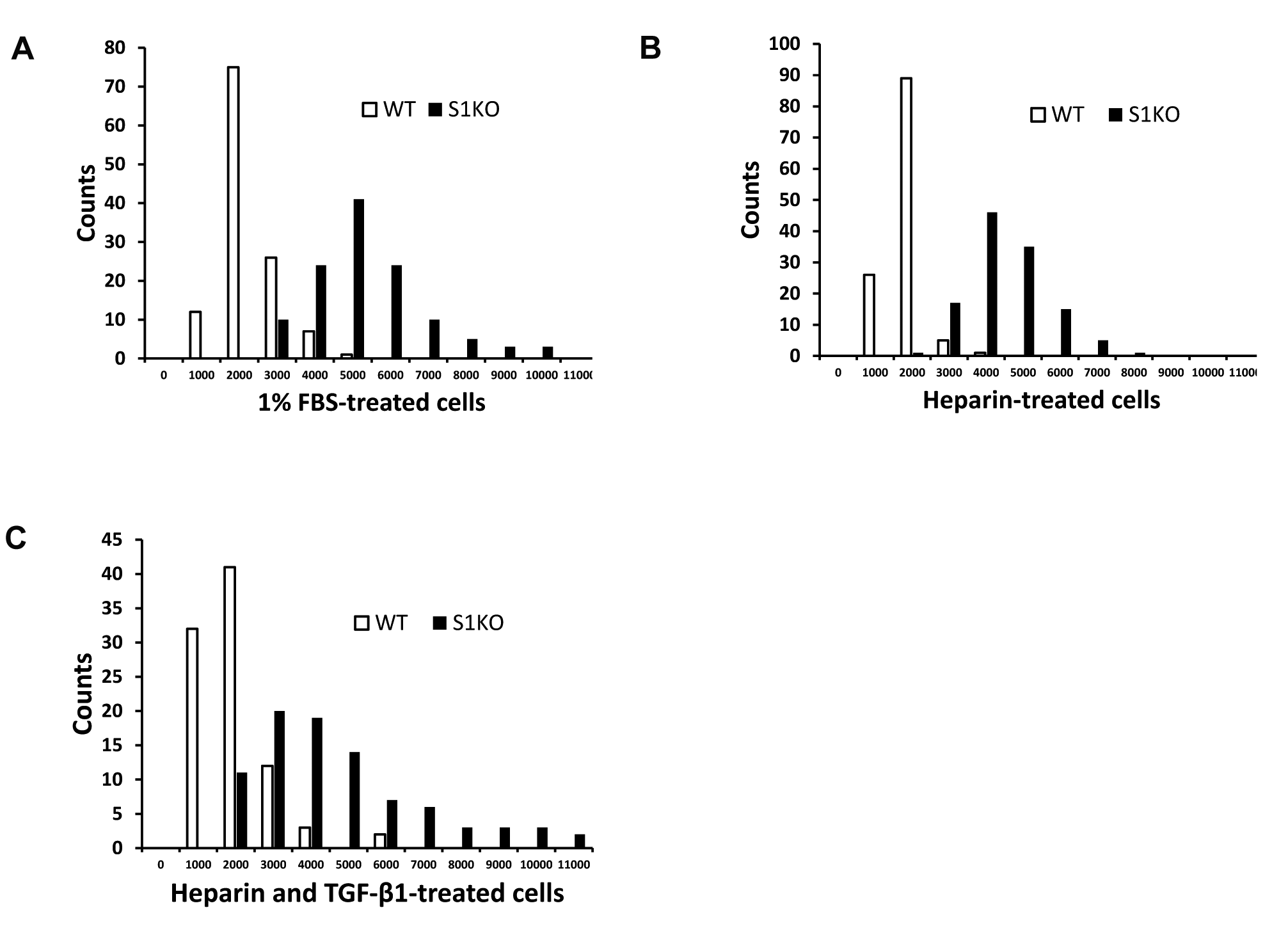

Supplement: Figure S2 — Histograms of cell areas for (A) 1F-treated vSMCs (control cells), (B) heparin-treated vSMCs, and (C) heparin and TGF-β1-treated vSMCs. Binning in the x-axis is area in µm2. (TIF) [file pone.0089824.s002.tif]

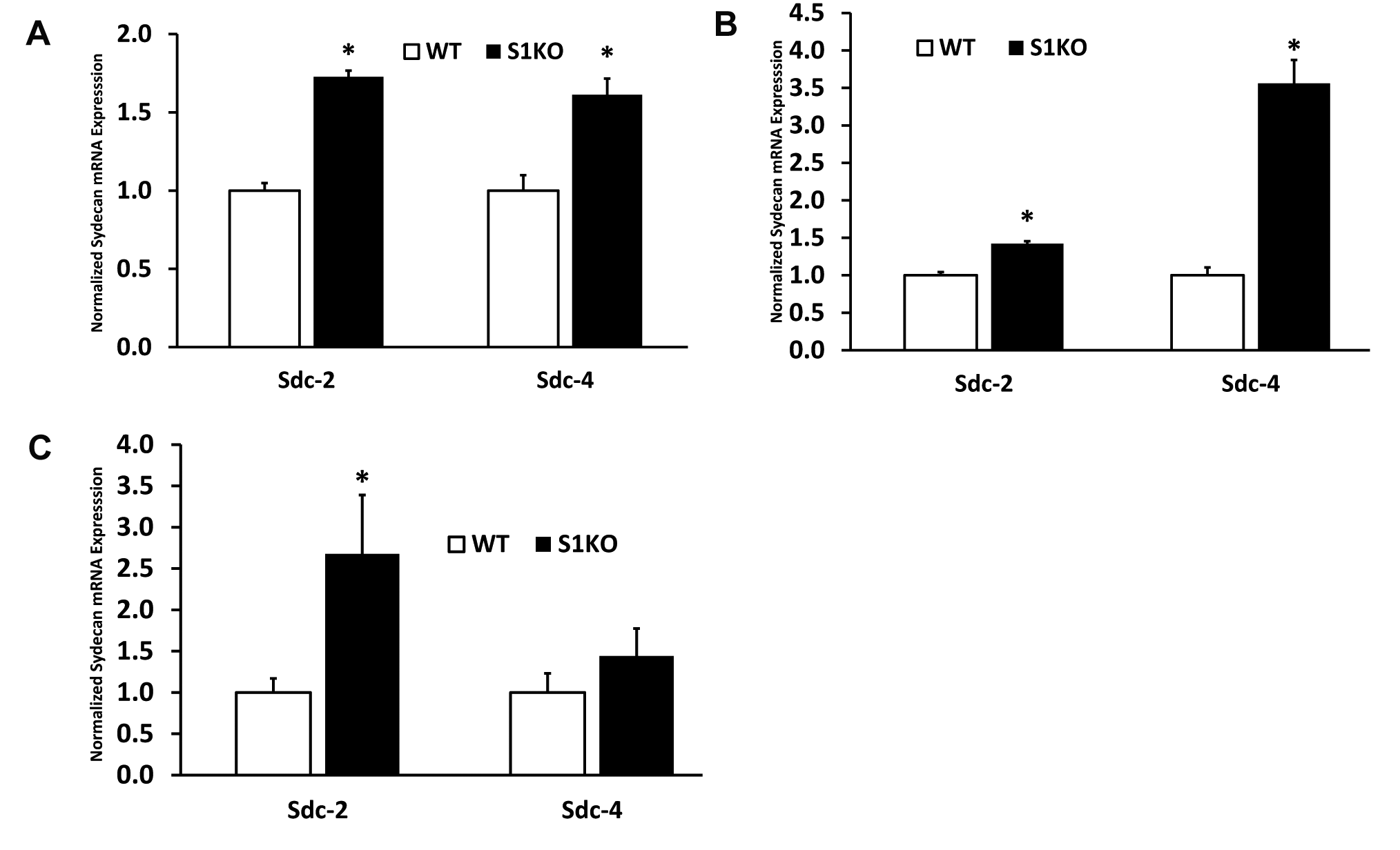

Supplement: Figure S3 — Gene expression for syndecan-2 (sdc-2) and syndecan-4 (sdc-4) in vascular smooth muscle cells (vSMCs) isolated from wild type (WT) and syndecan-1 knockout (S1KO) mice. Cells were treated with (A) control (1% FBS), (B) medium containing 30 µg/mL heparin, and (C) medium containing 30 µg/mL heparin and 5 ng/mL TGF-β1. *Statistically significant difference with WT group under similar culture conditions (p<0.05). (TIF) [file pone.0089824.s003.tif]
