# Supplementary material for: Syndecan-1 Regulates Vascular Smooth Muscle Cell Phenotype
Source: PLoS One. 2014 Feb 25;9(2):e89824. doi: 10.1371/journal.pone.0089824 (PMC3934950; doi:10.1371/journal.pone.0089824)
Supplement: Table S1 — (DOCX) [file pone.0089824.s004.docx]

| **Supplementary Table 1.** *Mouse and human primers used for real time RT-PCR experiments*  **A**  **C** | | |
| --- | --- | --- |
|  | **Forward Primer Sequence (5'-3')** | **Reverse Primer Sequence (5'-3')** |
| ***Mouse*** |  |  |
| GAPDH | CGACTTCAACAGCAACTC | TATTCATTGTCATACCAGGAA |
| α-SMA | AAGTATCCGATAGAACAC | AAACATAATCTGGGTCAT |
| Calponin | TCATTCTTTGCGAATTTATC | GGACTGAACTTGTGTATG |
| Transgelin | CTGGTTTATGAAGAAAGC | TTCTAACTGATGATCTGC |
| Desmin | TACAAGTCCAAGGTTTCA | TCGGTATTCCATCATCTC |
| MYH11 | AAGAGAATGAGAAGAAAGC | AGTGTATTCCTTCCTGAC |
| SMemb | GAGAAGAAACTGAAAGAAATC | TCTCCATCTGCTCCTTAT |
| Tissue Factor | GCGTTTAATTTAACTTGGA | TCGATCACTTATCTGTAC |
| Syndecan-1 | TCTGTCATCAAAGAGGTT | AAAGGTGAAGTCTTGTTC |
| Syndecan-2 | TCCAGTTCTGACAACATC | TTCTTCAGGTGACTCAGT |
| Syndecan-4 | TCTGGAGATCTGGATGAC | CTCAGGGATGTGGTTATC |
| ***Human*** |  |  |
| GAPDH | TATTCTCTGATTTGGTCGTA | ATGGCAACAATATCCACT |
| α-SMA | AGACTTCCGCTTCAATTC | CTGTTAGGACCTTCCCTC |
| Calponin | ACACAACTACTACAATTCC | TCTCTCCAAACTCTAACC |
| Smoothelin | TGAGGAGCTGATGACTATT | CTGATCCAGCATCTTGTC |
| MCP-1 | TTCCTCTTGAACCACAGT | CTTGCAAAGACCCTCAAA |
| ICAM-1 | GACTAAGCCAAGAGGAAG | CTCAGCATACCCAATAGG |
| VCAM-1 | CCTGCCATTGGAATGATAA | TGCTTCTACAAGACTATATGAC |
